# Multi-Drug Featurization and Deep Learning Improve Patient-Specific Predictions of Adverse Events

**DOI:** 10.3390/ijerph18052600

**Published:** 2021-03-05

**Authors:** Ioannis N. Anastopoulos, Chloe K. Herczeg, Kasey N. Davis, Atray C. Dixit

**Affiliations:** 1 Biomolecular Engineering, University of California, Santa Cruz, CA 95064, USA; ianastop@ucsc.edu; 2 Coral Genomics, Inc., 953 Indiana St., San Francisco, CA 94107, USA; chloe@coralgenomics.com (C.K.H.); kasey@coralgenomics.com (K.N.D.)

**Keywords:** adverse events, real world evidence, neural networks, graph convolution, FDA FAERS, UK Biobank

## Abstract

While the clinical approval process is able to filter out medications whose utility does not offset their adverse drug reaction profile in humans, it is not well suited to characterizing lower frequency issues and idiosyncratic multi-drug interactions that can happen in real world diverse patient populations. With a growing abundance of real-world evidence databases containing hundreds of thousands of patient records, it is now feasible to build machine learning models that incorporate individual patient information to provide personalized adverse event predictions. In this study, we build models that integrate patient specific demographic, clinical, and genetic features (when available) with drug structure to predict adverse drug reactions. We develop an extensible graph convolutional approach to be able to integrate molecular effects from the variable number of medications a typical patient may be taking. Our model outperforms standard machine learning methods at the tasks of predicting hospitalization and death in the UK Biobank dataset yielding an R^2^ of 0.37 and an AUC of 0.90, respectively. We believe our model has potential for evaluating new therapeutic compounds for individualized toxicities in real world diverse populations. It can also be used to prioritize medications when there are multiple options being considered for treatment.

## 1. Introduction

Clinical trials are used to determine the efficacy and toxicity of medications in humans. Although effective in elucidating various acute responses to the pharmaceutical compound in question, clinical trials are inherently limited by representation bias, size, and duration [1,2]. Current methods of toxicity and drug testing are unable to predict adverse drug reaction (ADR) across diverse populations under conditions of chronic exposure [3,4]. Such ADRs are a significant global health issue that affects millions of people each year with and accounting for an estimated 17% of hospital readmissions [5,6,7,8].

One response to this inherent short-coming in predicting and preventing ADRs has been the Tox21 Program. Through collaborative efforts, the U.S. National Institute of Health (NIH), Federal Drug Administration (FDA), and Environmental Protection Agency (EPA) have come together to help promote the evolution of toxicological testing and achieve specific goals that would increase both acute and predictive testing capacities [9]. To increase the ability to understand toxicity effects via data-driven predictions, the program outlines key objectives that address current limitations in identifying rare idiosyncratic responses, characterizing non-genotoxic potential carcinogens, gaining further insight into Adverse Outcome Pathways for risk assessment, and other gaps in testing technology [9].

To fuel the large-scale studies geared towards advancing toxicological and predictive technology, scientists utilize centralized real-world evidence (RWE) databases that contain individual level records of adverse events and associated patient features. Such sources, include the FDA Adverse Event Reporting System (FAERS) dataset which has been standardizing post-market adverse event reports for over six years and the UK Biobank (UKBB), which links a large set of clinical variables (including medications) longitudinally to genetic information [5,10]. These databases compile relevant information with the intent to improve public health through innovation and discovery [11].

Machine learning may be able to fill the gaps outlined in the Tox21 program by creating models that are predictive, scalable, cost-effective, and adaptable. More specifically, computational methods that have been adapted to biomedical applications, such as toxicity testing, drug responses, and drug discovery, include approaches that leverage Morgan Fingerprints (Morgan FP), Graph Convolutional Networks (GCNs), and Neural Networks (NNs) for Deep Learning applications [12,13].

Such methods utilize structural characteristics of molecules as inputs to computational programs, which in turn, informs in vivo response predictions. Morgan FP, for example, represent key molecular substructures using an explicitly defined featurization. However, a limitation of this specific methodology is its inability to adaptively learn alternative representations that may be more adapted to a particular task [13].

Differing from traditional fingerprint representations, GCNs represent atoms, molecular connectivity, and bond characteristics in a graph-based format. The relationships between neighboring atom-level features that are most informative for a particular task can be learned as the network updates the weights connecting each of the graph convolutional layers [13]. Since featurization and task prediction happen simultaneously in GCN based models, they have significantly higher model complexity and typically require a large dataset in order to outperform traditional machine learning approaches based on traditional fingerprint representations. Alternatively, a pre-training strategy can be used where the model is first trained on a related task (on which a large dataset is available) and then subsequently fine-tuned [14].

Integrative approaches are required to not only enable accurate predictions, but also to address the need for real world applicability. In clinical practice, it is common for those with chronic disease to be on a regimen of multiple medications, which has a positive correlation with the occurrence of ADRs [15,16]. Not only are multiple medications required for patients with comorbidities, but a single illness may also commonly treated with more than one medication [17,18]. The Center for Disease Control reported that from 2013–2016, in the U.S. alone, 24% of the population reported using three or more medications, whereas 12.6% reported using five or more within the month preceding the survey [15].

While efforts have been made to create machine learning approaches and corresponding databases that capture new ADRs or drug-drug interactions [19], they are limited in their ability to generalize across larger sets. The computational costs and data required to model these higher order interactions scales exponentially with the number of drug interactions (i.e., n, n^2^, n^3^, for single drug, drug-drug, and drug triplet interactions respectively) and as such no existing method can flexibly learn interactions across all medications for patients on multiple medications. Additionally, incorporating other risk factors into the model such as demographic and clinical data can be important in obtaining the most accurate predictions and disentangling confounding risks [2,20,21].

In this paper, we discuss an integrative, precision medicine approach to multi-drug adverse event prediction. The strategy utilized seeks to fill translational gaps in current predictive methodologies.

## 2. Materials and Methods

### 2.1. Drug Name to Chemical Structure

Pubchempy (an open source Github repository) was used to convert drug names or active ingredient to isomeric SMILES representation. Simple text filtering for case and punctuation was performed on the drug name input.

### 2.2. Chemical Structure Featurization of Single Molecules

In our GCN, SMILES structures are featurized using functionality from RDkit (an open source Github repository). Specifically, atom-level features consisting of a one-hot encoding if the atom is either C, N, O, F, P S, Cl, Br, I, the atomic number, a one-hot coding of chirality, the atoms degree (number of neighboring atoms), formal charge, number of hydrogens, number of radical electrons, a one-hot encoding of hybridization, whether it is or is not aromatic, and whether it is or is not in a ring. Bond-level features including, conjugated status, a one hot encoding of bond type (single, double, triple, aromatic), and a one hot encoding of whether the bond is stereoisomeric. We chose to concatenate the bond-level features to the atom-level features by summing over all bonds directly connected to each atom. This resulted in a feature vector of length 42 for each atom. Finally, we constructed the connectivity matrix between atoms and loaded these features into a Pytorch Geometric Data object.

For cases in which a linear model was to be used as a comparator to the neural network architecture, SMILES structures were featurized into binary feature vectors of length 2048 using Morgan FP with radius 2 using the python package RDkit.

### 2.3. Extension to Multi-Drug Framework

For our GCN, the atom-level connectivity matrices for each molecule were connected in a block diagonal manner with atom-level and bond-level features being adjoined directly.

For use in the linear model comparisons when patients were taking multiple medications, the maximum value of each element of the Morgan fingerprint across all medications was used as the corresponding featurization for the linear model (again resulting a vector of length 2048).

### 2.4. Linear/Logistic Regression

For continuous variables, such as predicting hospitalization in the UKBB dataset, a linear regression with an L^2^ norm penalty (Ridge regression) was used as a comparator model (sklearn’s Ridge module with default parameters).

For discrete variables, such as predicting death or outcome labels in the FAERS dataset a comparable model using sklearn’s logistic regression (with default parameters) was used.

### 2.5. Neural Network/Graph Convolutional Neural Network

For non-drug features, a simple neural network was constructed with the following form:

1. Linear layer transforming the feature vector into a hidden dimension (100 in our model)
2. Rectified linear unit (ReLU) transform
3. Batch normalization
4. Fully connected linear layer transforming hidden dimension to hidden dimension
5. ReLU transform
6. Linear layer transforming hidden dimension to target dimension

For medication associated features, the following architecture was used based roughly on [14,22]:

1. GINConv (graph isomorphism) layer feature vector into a hidden dimension (100 in our model)
  - This model performs uses a small neural network to map input atom-features to the output dimension taking into account neighboring atoms
2. Rectified linear unit (ReLU) transform
3. Batch normalization
4. Four additional layers as in 1–3 above

Medication features are aggregated using pooling operators including Set2Set [23], global_max_pool, and global_mean_pool, available in the PyTorch Geometric library.

For the combined model, the outputs of the architectures described above are concatenated for the relevant feature subset.

### 2.6. Model Evaluation

In all cases, 5-fold cross validation is performed to evaluate model performance.

### 2.7. Feature Attribution/Importance

To evaluate the importance of individual features within the combined neural network architecture the Integrated Gradients method [24] within the Captum library for PyTorch was used. The sum of all gradients for each feature across patients is used as an estimate of feature importance.

### 2.8. UK Biobank

UK Biobank (UKBB) contains deep genetic and phenotype information on approximately 500,000 individuals from across the United Kingdom who were aged 40 to 69 at recruitment [10]. Our data were resourced under Application Number 5424. It is available to researchers pending confirmation of their institutional affiliations by their approval committee and payment of any applicable fees.

Medication and health supplements data (Data Field: 20,003) were coded using 6745 categories (Data coding 4) which were mapped to their corresponding active ingredient follow steps similar to those in [11]. This active ingredient was used to obtain SMILES stings and featurization as described above.

Clinical features for each patient were extracted from ICD10 codes (Data Field: 41,202). PCA was performed across all patients to reduce the dimensionality and the scores were extracted as a representation of the “clinical status” of each patient.

A summary of the key subsets of the UKBB dataset that we reference in this work is described in Table 1.

### 2.9. FDA FAERS

We captured a total of 8,224,912 unique cases in the FAERS database spanning the years between 2014–2020 (through Q3 2020). The data may be readily accessed through the FDA’s online portal. The data were further filtered by the reported role the drug played in the adverse event report, which is characterized by the physician. We only selected drugs that were characterized as primary suspect drugs, secondary suspect drugs, or suspected interacting drugs (PS, SS, or I in the DRUG file), meaning they may have played an important role in the adverse event. Finally, we performed filtering to remove potentially duplicated entries for cases in which the same combination of sex, weight, age, and medications appeared more than once.

A summary of the key subsets of the FAERS dataset that we reference in this work is described in Table 2.

## 3. Results

**Figure 1:**
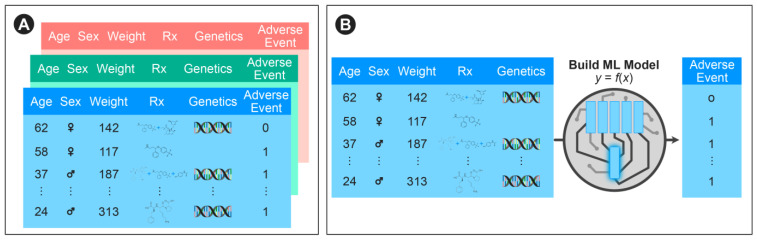
Overview of our approach: (**A**) Integration of multiple real world evidence databases including demographic, medication, and genetic information; (**B**) A machine learning model to predict adverse events is constructed.

We sought to construct a machine learning framework that could incorporate vast (but often disparate and filled with missing data elements) RWE databases to predict adverse events (Figure 1). We imposed the requirement that the model be able to flexibly model patients who are on multiple medications without being explicitly constrained to pairwise drug-drug interactions or those previously described.

**Figure 2:**
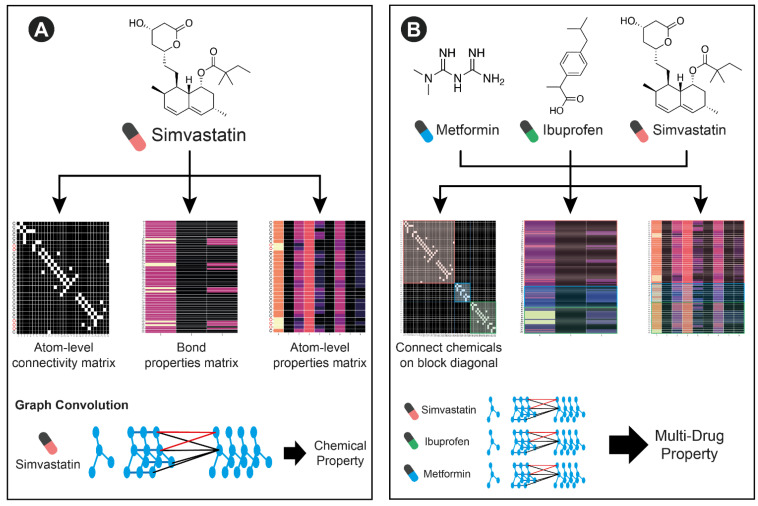
An overview of the multi-drug GCN architecture: (**A**) A standard GCN applied to a chemical structure creates bond and atom-level features, and an atom-level connectivity matrix to describe the molecule. Graph convolutions are performed to learn new feature representations that learn local structures that can be used to predict chemical properties (**B**) Our multi-drug GCN architecture concatenates the bond and atom- level features and creates a block diagonal connectivity matrix that represents the set of molecules an individual is taking. In a generalization of the single molecule GCN, the multi-drug GCN aggregates information from local structures across all molecules to predict multi-drug properties. We highlight the featurization of an example patient currently taking simvastatin (red pill), ibuprofen (green pill), and metformin (blue pill).

In order to model the variable number of medications that any patient may be taking, we leverage the graph-based featurization of molecules that GCNs can learn (Figure 2). A single chemical can be represented by the connectivity between atoms, atom-level features, and bond-level features. By concatenating atom level connectivity matrices in a block diagonal format, and simply concatenating atom and bond-level features, multiple molecules can be featurized together. This concatenation represents a collection of disjoint subgraphs. Since there are no connections between different molecules in the connectivity matrix, the GCN operations will not incorporate information across molecules. However, subsequent fully connected operations can learn from the collection of extracted features. With a sufficiently large training dataset, this architecture is able to learn new chemical features and interactions between them that predict multi-drug properties including adverse events.

In order to flexibly model other available individually predictive features (such as age, sex, weight, and genetics), we create separate compact neural networks that learn representations of these features. Finally, the learned representations across each small neural network and the GCN can be combined in a final set of neural network layers to predict the patient-level variable of interest.

### 3.1. Predicting Adverse Events in the UK Biobank Dataset

**Figure 3:**
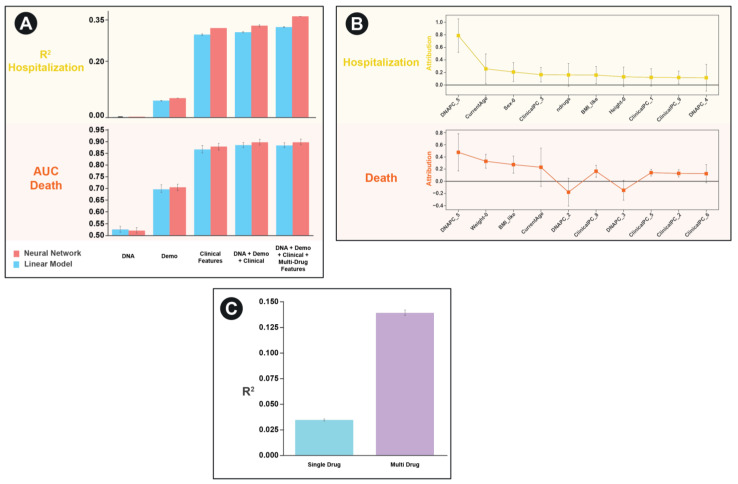
Predictive utility of various features and model architectures for predicting adverse events in the UKBB dataset: (**A**) Results for hospitalization (top) and death (bottom). Red bar corresponds to neural network architecture, blue bar corresponds to linear model. *Y*-axis is an R^2^ measure of model performance on predicting log10 (hospitalization + 1) (top) and AUC for predicting death (bottom). *X*-axis contains various combinations of features used in the model. Error bars correspond to 95% confidence interval derived from bootstrapping on 5-fold cross-validation (each fold contains 58,312 patients). (**B**) Feature weights (attributions) for each the top 10 most important non-drug structure features in the integrated model for predicting hospitalization in the UKBB dataset (top) and death (bottom). Error bars correspond to ±1 s.d. (**C**) Bar plot comparing results of using single drug features to using multi-drug features alone for predicting hospitalization. Error bars correspond to 95% confidence interval derived from bootstrapping on 5-fold cross-validation (each fold contains 42,114 patients).

In order to test the performance of our framework, we applied it to the UK Biobank dataset on two separate tasks: predicting the number of hospitalizations a patient experienced and predicting whether an individual has died. We were particularly interested in characterizing the relative importance of each of the features and any nonlinear interactions between features. As such we create separate models that contained each of the individual features as well as a combined model containing all features. To benchmark the performance of our approach, we contrast the neural network performance with that of a linear model (which would have limited ability to discern interactions between feature sets) (Figure 3A).

For both the task of predicting hospitalization and death in the UKBB, we found that clinical features (based on PCA scores of ICD10 codes) to be the most predictive individual feature set. For hospitalization, but not death, we find that the neural network architectures significantly outperformed the simple linear model for every feature set except the genetic principal components by themselves. Similarly, for hospitalization, but not death, we find a combined model including multi-drug GCN features significantly improves the predictive performance of the model compared to one without those features (R^2^ 0.364 vs. 0.331, *p* = 0.00004, Figure 3A).

As a final evaluation of feature importance in the combined model, we use the Integrated Gradients approach to assess contributions of the non-drug features [24]. Surprisingly, we find that despite the fact that the both DNA and demographic features had relatively low predictive performance individually, they were amongst the most predictive features in the combined model (Figure 3B).

To highlight the improvements made possible by our multi-drug framework compared to a single-drug framework, we revaluated performance on the task of predicting hospitalization for the subset of patients who are on 2 or more medications. We compare the performance of a model that only considers one randomly selected drug per patient to a model that considers all drugs (Figure 3C). We find a highly significant improvement in model performance (R^2^ of 0.035 vs. 0.14, *p* < 10^−10^).

### 3.2. Predicting Adverse Events in the FDA FAERS Dataset

We next sought to apply our framework to the FDA FAERS dataset. It contains a larger volume of data and a more targeted set of adverse event labels. Specifically, we attempted to predict the outcomes codes using a similar set of features to those available in the UK Biobank (except for DNA/genetic features which are not available in FAERS).

**Figure 4:**
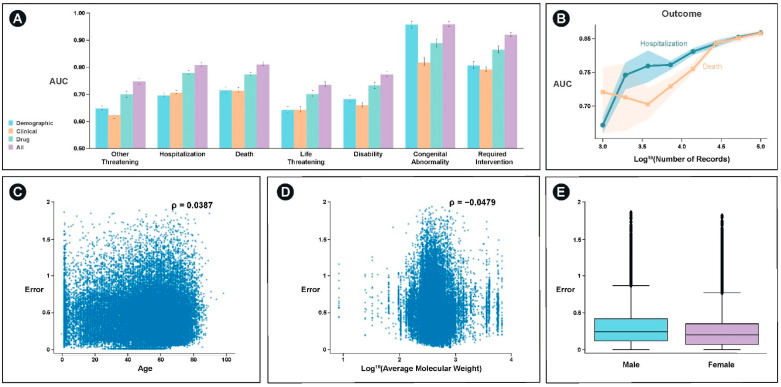
Performance comparisons on the FAERS dataset. (**A**) Predictive utility of various features and model architectures for predicting adverse events in the FAERS dataset. *X*-axis labels correspond to adverse event categories for a particular case. *Y*-axis is the AUC at predicting each of the labels. Colors correspond to various feature subsets tested. Error bars correspond to 95% confidence interval derived from bootstrapping on 5-fold cross-validation (each fold contains 28,682 records). (**B**) Power analysis demonstrating improvement in performance as a function of the number of patient records examined. Blue corresponds to hospitalization model performance and orange corresponds to performance of model predicting death. *X*-axis is log10 (number of records) *Y*-axis is AUC. Shaded error region corresponds to 95% confidence interval derived from bootstrapping on 5-fold cross-validation in a subsampled dataset corresponding to the *X*-axis location. (**C**) Plot demonstrating relationship between model error across all outcomes and age, (**D**) average molecular weight of drugs patient is taking, and (**E**) patient sex.

With the exception of congenital abnormality, which can be significantly predicted with demographic information such as age, the best single feature set for predicting the majority of outcomes was drug structure specific features (Figure 4A). For most categories, an integrated model of demographic, clinical, and drug structure significantly outperformed any of the individual feature set models. These categories included hospitalization (*p* < 10^−5^) and death (*p* < 10^−5^).

We examined the extent to which model performance would be expected to improve through the incorporation of additional data and find that the model would continue to improve for both the prediction of hospitalization and death (Figure 4B). Additionally, we examine the extent to which the model performs better or worse as a function of the covariates we used. We find weak, but significant relationships between age, average molecular weight, and sex and model error (*p* < 10^−6^) (Figure 4C–E).

## 4. Discussion

The two datasets analyzed in this paper have contrasting strengths and weaknesses. The UK Biobank includes deep genetic and phenotype information to compare the relative predictive performance of a wide range of well annotated features, but is generally limited to adults 40 years and older in the United Kingdom. It does not have a well-curated a collection of adverse event labels and, as such, surrogate labels such as hospitalization or death were used in this paper. In contrast FDA FAERS collection, is solely focused on adverse events with detailed event labels, however, the database contains selection bias against patients who did not suffer any adverse events on a medication. As such, a predictor built on FDA FAERS will overestimate the likelihood of a particular patient in the general population on a particular combination of medications having an adverse event without performing an additional calibration for how widely those particular medications are prescribed.

There are several promising directions for expanding upon and improving the approach described in this paper. These range from feature expansion for the representation of each atom (including aspects such as drug route of administration and dosage) in each chemical to optimization of the model architecture. One particularly promising area is the incorporation of convolutions which incorporate bond features and the spatial relationships between atoms [25,26]. 

The Integrated Gradients approach that we used can also be used to increase model interpretability on drug features. Specifically, the relative importance of particular chemical motifs (and interactions between motifs across medications) that drive the prediction a particular individual to experience an adverse event can be visualized [24].

We also note several limitations of the work we present here. Modification of model architecture would likely be required to incorporate and model the impact of biologic therapies. Additionally, for the FAERS dataset, we filtered to around 3% of the overall dataset, this limited dataset may have reduced the ability of the GCN approach to learn improved featurizations. As such, we could either use less strict filtering or pre-train the GCN using other datasets such as the Tox21 Data Challenge or UKBB data sets.

Despite the limited performance of genetic features as standalone predictors of ADRs, we were encouraged by the feature importance of several genetic principal components in the combined model to predict hospitalization in the UKBB dataset. As such we explored using a more comprehensive genetic feature set and developed a companion manuscript, which describes a more thorough variant level prediction of the genetic basis of ADRs across the millions of genetic variants present in the UKBB.

Finally, we highlight two specific examples to illustrate situations in which our model performs poorly and when it performs well. In the first example, we describe the case of a 30-year-old female on Nexplanon who experienced a hospitalization and related life-threatening event that our model failed to predict. We find multiple similar cases of patients on Nexplanon or Nuvaring (implantable birth control medications that the model performed poorly on (there are 659 such cases in our FAERS dataset, and we find they have a 32% higher error than other cases, *p* < 10^−4^). We hypothesize that this is due to the route of administration not being a component of our model (i.e., pill, infusion, eluting implantable device, etc). In our second example, we highlight the case of a 35-year-old female taking multiple medications who is likely immunocompromised on medications for multiple infections and HIV antiretrovirals whose four adverse events were predicted almost perfectly (difference between actual adverse event outcomes and predicted probabilities was 0.82 out of 7).

## 5. Conclusions

In this work, we compare the relative predictive utility of demographic, genetic, clinical, multiple drug structures, and the integration of these features to predict adverse outcomes in real world evidence databases including the UKBB and FAERS dataset (Table 3).

In the UKBB, we find that in many cases the incorporation of a neural network framework significantly improved predictive performance relative to a standard linear model suggesting the presence of nonlinear interactions between features. We also find that in an integrated model of all features, which outperformed and of the single feature models for hospitalization, demographic and genetic features had significant weights despite not having strong individual level performance.

Similarly, in the FAERS dataset we find that a combined model of demographic, clinical, and multi-drug feature sets is able to outperform any individual feature set for key outcomes like hospitalization and death. This result suggests a role for personalized medicine approaches to predictive toxicology that incorporate patient specific and multi-drug structure features into joint models.

As part of this work we outline and implement a multi-drug GCN framework that is able to flexibly incorporate the variable numbers of medications that real-world patient populations are taking. Built on a deep neural network architecture and deployed on GPU frameworks, it has the potential to rapidly learn complex interactions from growing databases of real-world evidence.

Overall, we believe that these methods will facilitate more accurate predictive personalized toxicology efforts in the future.

## Figures and Tables

**Table 1:** Summary of key parameters of UKBB dataset.

Feature	Value
Number of patients selected after filtering	291,560
Average number of medications per patient	3.2
Demographics	Age, Sex, Weight, Height, BMI, and the number of drugs the patient is taking
DNA	Scores from first five genetic PCA components from UKBB—Data-Field 22,009 [10]
Clinical	Scores from first 10 PCA components of ICD10 codes—Data-Field 41,202 (see description above)
Drug structure	For linear model, the maximum of the Morgan Fingerprint is used to featurized multi-drug features, for the GCN, the featurization is flexibly learned during model training
Hospitalization	Log10 (hospitalizations documented + 1)—Data-Field 41,235
Death	Based on Data-Field 40,000

**Table 2:** Summary of key parameters of FAERS dataset.

Feature	Value
Number of cases selected (after filtering)	143,412
Average number of medications per case	1.5
Demographics	Age, Sex, Weight, Reporting country
Clinical	Individual presence or absence for the top 200 indications for which drugs were prescribed in the entire FAERS database
Drug structure	For the GCN, the featurization is flexibly learned during model training

**Table 3:** Summary and support of key findings in each of the two datasets examined.

Attribute	UKBB	FAERS
Neural network outperforms linear model for individual features	For hospitalization across all features except genetic principal components, but not death	For most categories except congenital abnormality, and disability and most models except those only involving clinical features
Combined multi-drug model improves performance relative to other feature sets	For hospitalization, but not death	For both hospitalization and death
Most important single feature	Clinical ICD10 features	Multi-drug features

## Data Availability

Restrictions apply to the availability of the UK Biobank dataset and it is available through an application process. The FDA FAERS dataset is publicly available. This data can be found here: https://www.fda.gov/drugs/questions-and-answers-fdas-adverse-event-reporting-system-faers/fda-adverse-event-reporting-system-faers-latest-quarterly-data-files (accessed on 14 December 2020).
