# Correction: Anastopoulos et al. Multi-Drug Featurization and Deep Learning Improve Patient-Specific Predictions of Adverse Events. *Int. J. Environ. Res. Public Health* 2021, *18*, 2600

**DOI:** 10.3390/ijerph19074216

**Published:** 2022-04-01

**Authors:** Ioannis N. Anastopoulos, Chloe K. Herczeg, Kasey N. Davis, Atray C. Dixit

**Affiliations:** 1 Biomolecular Engineering, University of California, Santa Cruz, CA 95064, USA; ianastop@ucsc.edu; 2 Coral Genomics, Inc., 953 Indiana St., San Francisco, CA 94107, USA; chloe@coralgenomics.com (C.K.H.); kasey@coralgenomics.com (K.N.D.)

In the original publication [1], there was a mistake in the colors associated with Figure 4A. The colors of the boxplots were incorrect.

The authors apologize for any inconvenience caused and state that the scientific conclusions are unaffected. The original publication has also been updated.

## Figures and Tables

**Figure 4:**
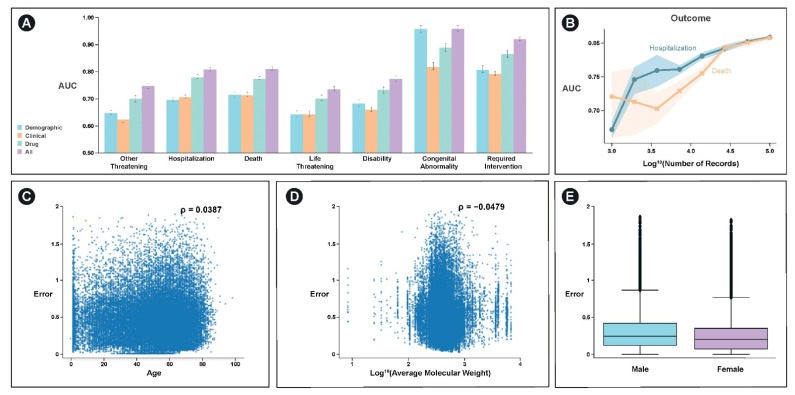
Performance comparisons on the FAERS dataset. (**A**) Predictive utility of various features and model architectures for predicting adverse events in the FAERS dataset. *X*-axis labels correspond to adverse event categories for a particular case. *Y*-axis is the AUC at predicting each of the labels. Colors correspond to various feature subsets tested. Error bars correspond to 95% confidence interval derived from bootstrapping on 5-fold cross-validation (each fold contains 28,682 records). (**B**) Power analysis demonstrating improvement in performance as a function of the number of patient records examined. Blue corresponds to hospitalization model performance and orange corresponds to performance of model predicting death. *X*-axis is log10 (number of records) *Y*-axis is AUC. Shaded error region corresponds to 95% confidence interval derived from bootstrapping on 5-fold cross-validation in a subsampled dataset corresponding to the *X*-axis location. (**C**) Plot demonstrating relationship between model error across all outcomes and age, (**D**) average molecular weight of drugs patient is taking, and (**E**) patient sex.
